# Diversity, Equity, and Inclusion in Gerontological Research Mentoring and Methodologies

**DOI:** 10.1093/geroni/igab046.908

**Published:** 2021-12-17

**Authors:** Ronica Rooks, Chivon Mingo, Chivon Mingo

## Abstract

With a rapid increase in our nation's diversity and in particular the diversity of the aging population, research focused on the well-being and quality of life for all older adults is imperative. Within GSA, The Minority Issue in Gerontology Advisory Panel is charged with providing support to the membership that ultimately will yield an increase in the quantity and quality of research related to minority aging issues. Therefore, understanding best practices for minority-focused gerontological research and gerontological education curriculum is warranted. The advancement of the field is predicated on the ability to have trained professionals with skills and competencies that effectively meet the needs of a diverse aging population. This symposium will include a presentation highlighting practical strategies for strengthening gerontology research by intentionally incorporating anti-racist methodological approaches. The second presentation will consist of recommendations on how to support, promote, and advance gerontology education in a manner that increases the diversity of those pursuing a research or an applied career in this area of study. Presenters will share an overview of the literature, findings from program implementation focus groups, and recommendations for tailoring strategies to fit your intended audience. This session will prove beneficial as we make strides to ensure that diversity, equity, and inclusion remain a core value and an inherent practice of all gerontology professionals.

